# Caregiver-child interaction and early childhood development among preschool children in rural China: the possible role of blood epigenome-wide DNA methylation

**DOI:** 10.1186/s12864-025-11406-2

**Published:** 2025-04-01

**Authors:** Mengna Wei, Rui Chang, Chunan Li, Yanfen Jiang, Jianduan Zhang

**Affiliations:** 1 Department of Maternal and Child Health, School of Public Health, Tongji Medical College, Huazhong University of Science and Technology, Wuhan, Hubei, China; 2 Division of Child Healthcare, Department of Pediatrics, Tongji Hospital, Tongji Medical College, Huazhong University of Science and Technology, Wuhan, Hubei, China; 3 Key Laboratory of Environment and Health, Ministry of Education & Ministry of Environmental Protection, and State Key Laboratory of Environmental Health (Incubation), School of Public Health, Tongji Medical College, Huazhong University of Science and Technology, 13 Hangkong Road, Hubei 430030 Wuhan, China

**Keywords:** Early childhood development, Caregiver-child interaction, Epigenome-wide DNA methylation study, Preschool children, Epigenetics, Rural China

## Abstract

**Background:**

While the association between caregiver-child interaction and early childhood development (ECD) has been observed, the underlying biological mechanism remains to be elucidated.

**Objective:**

This study aimed to examine the potential role of epigenome-wide DNA methylation in the association between caregiver-child interaction and ECD among preschool children living in rural China.

**Methods:**

This study was conducted in a rural area in Central China. ECD was evaluated with the Gesell Development Diagnosis Scale (Chinese version), yielding a developmental quotient (DQ), i.e. global neurodevelopmental score (NDS). Caregiver-child interaction was assessed using the Brigance Parent-Child Interaction Scale. Of the 171 children aged 3–6 years who participated in ECD assessment and blood samples collection, a total of 64 were selected for epigenome-wide association study with Illumina Infinium MethylationEPIC v1.0 BeadChip array (850 K). The linear regression model in the R package “CpGassoc”was applied to identify CpG sites associated with global NDS and caregiver-child interaction. The causal inference test (CIT) was utilized to explore the potential mediation effect of DNA methylation.

**Results:**

Our epigenome-wide DNA methylation analysis revealed 844 CpG sites significantly associated with children’s global NDS (*P*_FDR_<0.05), while no CpG sites were found to be directly related to caregiver-child interaction after FDR correction. Mediation analysis indicated that 395 CpG sites mediated the association between caregiver-child interaction and children’s ECD before FDR correction; and among the genes with top 20 CpG sites, genes *CFAP45* (cg07740897), *PCDH9* (cg20666533), *LAMC3* (cg14447608), *FAM19A5* (cg13192640), *PRKG1* (cg09071556), *PLEKHG5* (cg05151739), *TCERG1* (cg09189322), and *MTRR* (cg08075506) have been reported to be associated with neurodevelopment and related diseases.

**Conclusions:**

Blood DNA methylation may mediate the association between caregiver-child interaction and ECD in preschool children. This provides population-level epigenetic evidence supporting parenting interventions for vulnerable preschool children who experience poor caregiver-child interaction, aiming to ensure optimal early development potential. However, future studies in diverse populations are needed to validate these findings.

**Supplementary Information:**

The online version contains supplementary material available at 10.1186/s12864-025-11406-2.

## Introduction

Early childhood development (ECD) encompasses the progression of physical, cognitive, social-emotional, motor, and language skills, laying the foundation for an individual’s lifelong health, well-being, and success [1]. In low- and middle-income countries (LMICs), it is estimated that 43% (approximately 250 million) of children under five are not on track to fulfill their developmental potential, with China alone accommodating approximately 17.43 million affected children [2]. The consequences of inadequate early development are profound, including chronic disease, education underachievement, diminished annual income, and even inter-generational effects [2, 3], posing a significant public health concern [2]. Developmental delay during early childhood is attributed to the complex interplay of multiple factors, including genetic predisposition and environmental influences, which mutually shape the developmental process [4, 5]. Among environmental exposures, caregiver-child interaction plays a critical role [6, 7]. Extensive research has shown the transformative impact of caregiver-child interaction. For example, findings from the Shanghai Maternal-Child Pairs Cohort indicated that sustained exposure to responsive caregiving significantly reduced the risk of suspected developmental delay [7]. Similarly, improvements in the quality of caregiver-child interaction within institutional settings were associated with substantial gains in the physical, cognitive, and social-emotional development of children [8]. Poverty is often a precursor to suboptimal caregiver-child interaction [9]. Children living in resource-limited areas are frequently exposed to inadequate caregiver-child interaction, which increases their vulnerability to developmental delays. In our earlier investigation conducted within the same county, we found that a significant proportion (35.2%) of children aged 0–6 years experienced suboptimal caregiver-child interaction [10]. Importantly, those children who experienced poor caregiver-child interaction were found to be at an elevated risk of neurodevelopmental delays [β = -5.2, 95%CI (-10.0, -0.4)] and social-emotional delay [β = 10.0, 95%CI (6.9, 13.1)] [10], despite the high prevalence of suspected neurodevelopment delay and social-emotional delay within this demographic [6]. In addition, in poverty-stricken regions of Shaanxi and Guizhou Province, China, inadequate caregiver-child interaction has been identified as a contributing factor to suspected ECD delay in children under three years [11]. The growing body of evidence emphasized the important role of caregiver-child interaction in ECD, particularly within disadvantaged populations.

Despite the well-established influence of caregiver-child interaction on ECD, it is acknowledged that the underlying biological mechanism remains ambiguous. A growing body of literature suggests that environmental exposures might exert their biological effects through epigenetic mechanisms. DNA methylation, one of the most extensively investigated epigenetic modifications, involves the addition of a methyl group to the 5-carbon position of a cytosine residue, typically occurring at cytosine-guanine dinucleotides (CpG) [12]. As a potent regulator of gene expression, DNA methylation is regarded as the primary mechanism through which the lasting impacts of adverse environmental exposure are mediated [13]. For example, previous research in the UK suggested a negative association between CpG site methylation (e.g., cg27122725 annotated to *NR3C1*) and warmer, more positive maternal interaction with infants, while maternal neutral and hesitant behavior positively correlated with methylation at cg12466613 (annotated to *NR3C1*) [14]. Additionally, a review has documented an association between the quality of maternal caregiving and DNA methylation status in specific genes involved in socio-emotional development [15]. Although previous studies have indicated an association between caregiver-child interaction and DNA methylation, or between DNA methylation and neurodevelopment, few have examined the interplay between all three variables. A recent longitudinal study in the Netherlands focusing on the *NR3C1* gene reported that higher maternal sensitivity at infant five weeks of age predicted lower buccal DNA methylation levels at two NR3C1 CpG loci (cg21702128 and cg04111177). However, methylation levels at these loci did not mediate the effect of maternal sensitivity on children’s internalizing and externalizing behaviors [16].

While the aforementioned findings suggest a potential role of DNA methylation in linking caregiver-child interaction to ECD, existing human evidence is limited in several critical aspects. Firstly, previous research has predominantly focused on candidate genes, particularly those involved in the hypothalamic-pituitary-adrenal axis and serotoninergic system [17]. Fewer studies have explored epigenome-wide DNA methylation patterns. Secondly, the predominant focus of existing studies has been on analyzing DNA methylation in buccal or saliva samples, with only a limited number of investigations delving into DNA methylation in children’s peripheral blood. Given the marked tissue specificity of DNA methylation, the underrepresentation of blood samples in these studies restricts a comprehensive understanding of DNA methylation patterns. Thirdly, research examining the mediating role of DNA methylation in the association between caregiver-child interaction and ECD is limited. This knowledge gap is particularly pronounced in the context of rural China. Considering the genetic and environmental variations between Eastern and Western populations, the DNA methylation patterns observed in other ethnic groups may not be generalized to the Chinese population. In response to these research gaps, we conducted an EWAS in China to investigate the associations among blood DNA methylation changes, children’s ECD, and caregiver-child interaction. Importantly, our objective was to assess the mediating role of DNA methylation in the relationship between caregiver-child interaction and ECD.

## Methods

### Study population

The study was conducted between December 2021 and January 2022 in a former poverty county in Central China, involving 171 preschool children who participated in ECD evaluations. The inclusion criteria comprised residency in this area for more than six months, the absence of congenital disabilities, and having a primary caregiver proficient in verbal Mandarin communication. Participants who met these criteria were included in the neurodevelopment assessment, and blood samples were collected from them.

Ethical approval was obtained from the Ethics Committees of the Tongji Medical College, Huazhong University of Science and Technology in accordance with the Declaration of Helsinki. The legal guardians of all participants provided informed consent before enrollment.

### Assessment of early childhood development in preschool children

We accessed the ECD of children through the Chinese version of the Gesell Developmental Diagnostic Scale (GDDS; Chinese version) by trained staff, a well-accepted scale suitable for infants and children aged 0–72 months [18–20]. The GDDS assesses five specific domains: gross motor, fine motor, adaptive behavior, language, and social behavior. The result of each sub-domain is expressed as a developmental quotient (DQ), i.e. neurodevelopmental score (NDS), with a higher DQ score indicating better performance. The children’s total neurodevelopmental potential was represented by the average DQ score across the five domains, referred to as the global NDS [21–23]. A child with a global NDS below 85 was considered at risk for suspected developmental delay [21, 24]. Among the 171 participants, we identified 32 children with the most pronounced developmental challenges (global developmental score below 85) as cases. For the control group, we selected 32 children whose global developmental scores were within normal range (85 or higher), matched by age and sex with the cases. This matching was designed to minimize potential confounding variables influencing the association between DNA methylation patterns, caregiver-child interaction, and neurodevelopmental outcomes. We conducted EWAS on peripheral blood samples from these 64 participants using the Illumina Infinium MethylationEPIC v1.0 BeadChip array.

### Blood collection

Following the ECD assessment, trained medical staff collected 5ml blood specimens, which were stored in EDTA-treated tubes, and centrifuged at 3500r for 5 min. This centrifugation process enabled the separation of plasma and blood cells, which were subsequently collected in EP tubes and stored at -80 °C until used.

### Caregiver-child interaction assessment

We assessed caregiver-child interaction using the Chinese version of the Brigance Parent-Child Interactions Scale (BPCIS), as updated by Lai et al. [25, 26]. Considering the prevalence of migrant parents in our study area, we extended the scale’s focus to encompass the interactions between the primary caregiver and the child. The updated BPCIS is a concise tool designed to assess caregivers’ parenting behaviors and perceptions. It consists of 20 items that explore the frequency of interactions between primary caregivers and their children across various situations. Caregivers self-reported the frequency of these interactions, using response options including never, rarely, sometimes, often, or always. The updated BPCIS includes both positive and negative items. For negative items, we assigned scores of 5, 4, 3, 2, and 1 to responses from “never” to “always”, respectively. Conversely, for positive items, we used scores of 1, 2, 3, 4, and 5 for the same response options. We calculated total scores by summing across all items, providing an overall measure of caregiver-child interaction quality, with higher scores indicating more positive interactions. Caregiver-child interaction scores at or below 68 were considered as poor caregiver-child interaction. The reliability of this assessment, as indicated by Cronbach’s α (0.83), suggests an optimal internal consistency.

### Other information collection

We collected additional information concerning various children’s characteristics, including their sex (boy and girl), mode of delivery(vaginal delivery or cesarean section), preterm birth status (yes or no), sibling status (only child or non-only child), feeding mode at six month (exclusive breastfeeding, mixed feeding, or artificial feeding), parental education levels (primary school or below, secondary and high school, and university or above), and monthly household income (< 2000 RMB, 2000–3999 RMB, 4000–5999 RMB, and ≥ 6000 RMB). The sociodemographic characteristics of the 64 caregiver-child dyads did not exhibit significant disparities compared to those of the participants who were excluded from the study (Table S1).

### DNA methylation

DNA extraction was carried out in accordance with the manufacturer’s protocol using MagBead Whole Blood DNA Extraction Midi Kit III (Bio Teke Corporation, Beijing, China). The purified genomic DNA then underwent bisulfite conversion using the EZ DNA Methylation Gold kit (Zymo, CA, USA). Epigenome-wide DNA methylation was evaluated by the Illumina Infinium MethylationEPIC v1.0 BeadChip array, enabling the assessment to over 850, 000 CpG sites. Data pre-preprocessing normalization was performed using the "CHAMP" package in R [27]. The final DNA methylation analysis was performed utilizing a comprehensive set of probes, following rigorous quality control process: specifically, the exclusion of probes located on the X and Y chromosomes, as well as those overlapped with SNPs, had detection *P*-value > 0.01 in more than 10% of samples, exhibited bead counts of less than three, and were cross-reactive, or failed in one or more samples.

### Statistical analysis

Descriptive statistics were expressed as mean ± SD for continuous variables exhibiting a normal or near-normal distribution, while categorical variables were expressed as n (%). The Kruskal-Wallis test or chi-squared test was used to compare differences in demographic information across groups. The linear regression model in the R package “CpGassoc” was used to identify CpG sites associated with children’s global NDS and caregiver-child interaction [28]. Acknowledging the potential impact of cell type composition and batch effects on DNA methylation patterns, we have incorporated adjustments for the relative proportions of various cell types and batch effects in our analysis [29–31]. This is achieved using the smartSVA method and champ.runCombat function within the “ChAMP” package, respectively [29, 32]. For functional enrichment analyses, we employed the R “clusterProfiler” package that utilizes Gene Ontology (GO) and Kyoto Encyclopedia of Genes and Genomes (KEGG) databases [33]. The Benjamini–Hochberg False-Discovery-Rate (FDR) method was used to adjust for multiple testing, considering probes with a *P*_FDR_-value < 0.05 as significantly differentially methylated.

**Fig. 1:**
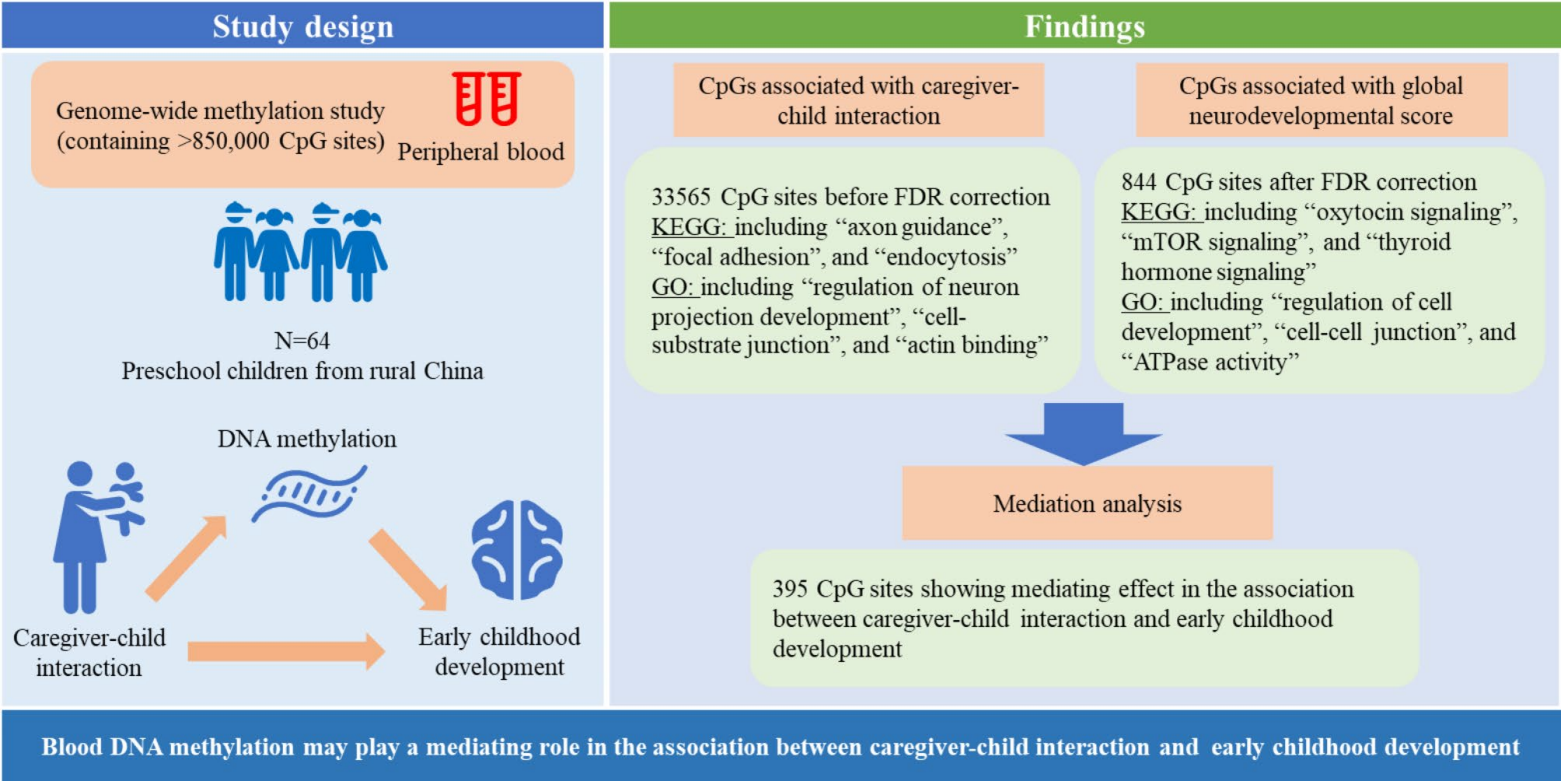
Summary workflow and results

The Causal inference test (CIT) was adopted to explore whether DNA methylation mediates the association of caregiver-child interaction and children’s global NDS [34, 35]. The CIT is a novel statistical framework that formalized existing notions of causal mediation into a hypothesis testing procedure [35]. We used the R software “cit” package to perform the causal inference test [34]. In our study, the CIT involves four linear regression models: Model 1 assesses the association of caregiver-child interaction with children’s global NDS; Model 2 examines the association of caregiver-child interaction with epigenome-wide DNA methylation level; Model 3 explores the association of epigenome-wide DNA methylation level with children’s global NDS; Model 4 investigates the association between caregiver-child interaction and children’s global NDS while adjusting the epigenome-wide DNA methylation level. In Model 4, we used the Bootstrap method for random sampling, repeating the modeling 1000 times to generate 1000 *P* values. A *P* value > 0.05 was considered irrelevant in this analysis. The mediation effect of DNA methylation was thought to exist if the *P* < 0.05 in Model 1, Model 2, and Model 3, whereas a *P* > 0.05 in Model 4. All the statistical analyses were performed with R software (version 3.6, R Foundation for Statistical Computing, Vienna, Austria). The flow chart summarizing the conducted analyses and primary findings is presented in Fig. 1.

## Results

### Characteristics of participants

Table 1 shows that, among the 64 participants, 56.2% (*n* = 36) were boys, 65.6% (*n* = 42) were delivered by cesarean section, 12.5% (*n* = 8) were born preterm birth, 25.0% (*n* = 16) were only children, and 50.0% (*n* = 32) were breastfeeding in the first six months after birth. Over half of the parents had obtained secondary and high school education levels, and the monthly household income was lower than 4,000 yuan.

**Table 1:** Basic characteristics of included participants

Characteristics	Level	Total (*n* = 64)	Preschool children with a global NDS below 85 (*n* = 32) No. (%)	Preschool children with a global NDS greater of 85 or higher (*n* = 32) No. (%)	Nominal *p* value
Child age (months), mean (SD), (months)	-	49.1 (7.5)	45.9 (5.4)	52.31 (8.0)	< 0.001
Sex of child	Boy	36 (56.2)	18 (56.2)	18 (56.2)	1.000
	Girl	28 (43.8)	14 (43.8)	14 (43.8)	
Preterm	No	56 (87.5)	28 (87.5)	28 (87.5)	1.000
	Yes	8 (12.5)	4 (12.5)	4 (12.5)	
Delivery mode	Vaginal delivery	22 (34.4)	5 (15.6)	17 (53.1)	0.004
	Cesarean section	42 (65.6)	27 (84.4)	15 (46.9)	
Only child	Yes	16 (25.0)	12 (37.5)	4 (12.5)	0.043
	No	48 (75.0)	20 (62.5)	28 (87.5)	
Feeding mode in the first six months	Basic breastfeeding	32 (50.0)	17 (53.1)	15 (46.9)	0.193
	Mixed feeding	23(35.9)	13 (40.6)	10 (31.2)	
	artificial feeding	9 (14.1)	2 (6.3)	7 (21.9)	
Paternal education	Primary school or under	4 (6.2)	0 (0.0)	4 (12.5)	0.018
	Junior/senior/vocational secondary School	56 (87.5)	28 (87.5)	28 (87.5)	
	College or above	4 (6.2)	4 (12.5)	0 (0.0)	
Maternal education	Primary school or under	8 (12.5)	2 (6.2)	6 (18.8)	0.206
	Junior/senior/vocational secondary School	55 (85.9)	29 (90.6)	26 (81.2)	
	College or above	1 (1.6)	1 (3.1)	0 (0.0)	
Household monthly income (yuan)	< 2000	13 (20.3)	5 (15.6)	8 (25.0)	0.788
	2000 ~ 3999	24 (37.5)	12 (37.5)	12 (37.5)	
	4000 ~ 5999	14 (21.9)	8 (25.0)	6 (18.8)	
	≥ 6000	13 (20.3)	7 (21.9)	6 (18.8)	
Father’s age (years)	-	33.3 (4.2)	32.87 (3.6)	33.81 (4.7)	0.382
Mother’s age (years)	-	31.16 (4.2)	31.52 (3.4)	30.81 (4.9)	0.510
Global NDS	-	86.8 (17.1)	72.1 (10.9)	101.4 (5.2)	< 0.001

global NDS: global neurodevelopmental score

### Epigenome-wide DNA methylation and ECD

The epigenome-wide DNA methylation analysis identified 844 CpG sites associated with children’s global NDS after FDR correction (*P*_FDR_<0.05, lambda = 9.43, adjusted lambda = 1.00). Of the top 20 CpG sites (Table 2), global NDS was negatively associated with methylation levels of cg02162544 and cg12625293 (both *P*_FDR_<0.05); and positively associated with methylation levels of cg03259342, cg22080978, cg08549311, cg13941555, cg13557301, cg10610099, cg20364213, cg01595505, cg06466407, cg14175218, cg02346006, cg06824812, cg00476580, cg17206054, cg12134970, cg13916351, cg00284493, and cg14286612 (all *P*_FDR_<0.05). To elucidate the functional implications of our findings, enrichment analyses were conducted on the genes annotated with methylated CpGs related to global NDS scores. KEGG pathway analysis detected several notable biological pathways, including the “oxytocin signaling pathway”, “mTOR signaling pathway”, and “thyroid hormone signaling pathway”. Simultaneously, GO analysis indicated significant enrichment in functions such as “regulation of cell development”, “cell-cell junction”, and “ATPase activity”. Further details are available in Tables S2-3.

**Table 2:** The top 20 differentially methylated CpGs associated with neurodevelopmental scores

CpG sites	Chr: location	Gene symbol	Gene region	Coefficient(β)	Nominal *p* value	*P*_FDR−value_
cg03259342	17: 79,518,195	FAAP100	Body-shore	0.00053	2.21E-07	0.01270
cg22080978	2: 27,428,049	SLC5A6	Body-opensea	0.00059	2.47E-07	0.01270
cg08549311	22: 43,568,240	TTLL12	Body-shore	0.00042	3.16E-07	0.01270
cg13941555	1: 2,565,265	MMEL1	TSS1500-opensea	0.00083	3.55E-07	0.01270
cg13557301	12: 110,838,588	ANAPC7	Body-shelf	0.00112	3.59E-07	0.01270
cg02162544	10: 72,163,814	EIF4EBP2	TSS200-island	-0.00046	3.59E-07	0.01270
cg10610099	19: 1,954,451	CSNK1G2	5’UTR-shore	0.00062	3.80E-07	0.01270
cg20364213	19: 19,730,261	PBX4	TSS1500-shore	0.00050	3.84E-07	0.01270
cg01595505	19: 39,905,835	PLEKHG2	Body-shore	0.00043	3.89E-07	0.01270
cg06466407	9: 128,312,369	MAPKAP1	Body-opensea	0.00052	4.04E-07	0.01270
cg14175218	11: 46,616,660	AMBRA1	TSS1500-shore	0.00106	4.04E-07	0.01270
cg02346006	17: 78,897,477	RPTOR	Body-shore	0.00045	4.25E-07	0.01287
cg12625293	2: 204,192,809	ABI2	TSS200-island	-0.00033	4.74E-07	0.01370
cg06824812	8: 1,731,480	CLN8	3’UTR-shore	0.00233	4.88E-07	0.01370
cg00476580	1: 150,207,583	ANP32E	TSS1500-island	0.00041	5.14E-07	0.01370
cg17206054	10: 553,484	DIP2C	Body-opensea	0.00087	9.14E-07	0.01711
cg12134970	3: 182,761,784	MCCC1	Body-opensea	0.00036	9.52E-07	0.01711
cg13916351	19: 50,101,736	PRR12	Body-shore	0.00055	9.61E-07	0.01711
cg00284493	5: 171,522,397	STK10	Body-opensea	0.00034	9.81E-07	0.01711
cg14286612	22: 43,845,465	MPPED1	Body-opensea	0.00058	9.84E-07	0.01711

Chr: Chromosome

### Epigenome-wide DNA methylation and caregiver-child interaction

Although no CpG sites were found to be associated with caregiver-child interaction after FDR correction (*P*_FDR_>0.05), a large number of CpG sites (33,565) were observed before FDR correction (lambda = 10.72, adjusted lambda = 1.00). Of the top 20 CpG sites (Table 3) before FDR correction, eight CpG sites (cg24001597, cg13619411, cg11395271, cg04996158, cg03498762, cg16391772, cg02337568 and cg18390596) were negatively associated with caregiver-child interaction; twelve CpG sites (cg09947274, cg13422701, cg13812840, cg15988632, cg12100302, cg10383568, cg10400804, cg14120224, cg10325933, cg18024368, cg05486832, and cg16144850) were positively associated with caregiver-child interaction. The KEGG analysis identified several biologically significant pathways, such as “axon guidance”, “focal adhesion” and “endocytosis”. Meanwhile, GO enrichment analysis revealed that these methylated genes were significantly enriched in “regulation of neuron projection development”, “cell-substrate junction” and “actin binding”. Additional information was found in Tables S4-S5.

**Table 3:** The top 20 differentially methylated CpGs associated with caregiver-child interaction

CpGs	Chr: location	Gene Symbol	Gene region	β	Nominal *p* value	*P*_FDR value_
cg24001597	18: 77,655,878	KCNG2	Body-shelf	-0.00139	9.08E-07	0.74212
cg13619411	2: 98,285,656	LINC01125	TSS1500-opensea	-0.00083	2.72E-06	0.99999
cg09947274	2: 27,439,915	C2orf28	3’UTR-shore	0.00160	1.66E-05	0.99999
cg11395271	2: 85,846,354	USP39	Body-shelf	-0.00097	2.22E-05	0.99999
cg13422701	12: 2,488,799	CACNA1C	Body-opensea	0.00162	2.41E-05	0.99999
cg13812840	16: 1,578,619	TMEM204	TSS200-shelf	0.00202	3.36E-05	0.99999
cg15988632	15: 65,904,714	VWA9	TSS1500-shore	0.00091	3.73E-05	0.99999
cg12100302	11: 70,265,910	CTTN	Body-opensea	0.00047	4.45E-05	0.99999
cg10383568	11: 6,589,761	DNHD1	Body-shelf	0.00191	4.81E-05	0.99999
cg10400804	16: 960,911	LMF1	Body-shore	0.00038	4.96E-05	0.99999
cg14120224	2: 219,575,507	TTLL4	TSS200-island	0.00043	5.38E-05	0.99999
cg04996158	19: 55,770,062	SAPS1	TSS200-island	-0.00027	5.50E-05	0.99999
cg03498762	12: 9,810,749	LOC374443	Body-opensea	-0.00097	7.04E-05	0.99999
cg16391772	18: 12,533,830	SPIRE1	Body-opensea	-0.00113	7.14E-05	0.99999
cg10325933	1: 23,074,675	EPHB2	Body-opensea	0.00114	8.56E-05	0.99999
cg18024368	6: 32,632,848	HLA-DQB1	Body-island	0.00283	8.76E-05	0.99999
cg05486832	19: 12,305,392	LOC100289333	TSS1500-shore	0.00198	9.35E-05	0.99999
cg16144850	14: 56,687,777	PELI2	Body-opensea	0.00166	9.44E-05	0.99999
cg02337568	12: 111,843,385	SH2B3	TSS1500-island	-0.00034	9.76E-05	0.99999
cg18390596	9: 117,691,764	TNFSF8	Body-opensea	-0.00064	9.96E-05	0.99999

Chr: Chromosome

### The potential mediating effect of epigenome-wide DNA methylation between caregiver-child interaction and ECD

CIT analysis identified 395 CpG sites with potential mediating effects in the association between caregiver-child interaction and children’s global NDS. In Model 1, a significant association was observed between caregiver-child interaction and children’s NDS (Nominal *P* < 0.05). Model 2 showed an association between caregiver-child interaction and epigenome-wide DNA methylation level before FDR correction. Similarly, Model 3 revealed an association between epigenome-wide DNA methylation level and children’s global NDS (all *P*_before FDR correction_ < 0.05). In Model 4, after adjusting the epigenome-wide DNA methylation level, the previously observed association between caregiver-child interaction and children’s global NDS became statistically insignificant (*Nominal P* > 0.05). These findings indicated that DNA methylation may act as a mediating factor in the association of caregiver-child interaction with children’s global NDS. The top 20 CpG sites (Table 4) identified as significant mediators in the association of caregiver-child interaction and global NDS were cg06261610, cg21055197, cg07740897, cg07111566, cg20666533, cg14447608, cg08665193, cg08482080, cg13192640, cg00608534, cg05621113, cg09071556, cg07546710, cg27297326, cg05151739, cg08084502, cg18086761, cg10142396, cg09189322, and cg08075506.

**Table 4:** The top 20 methylated CpGs playing mediating effects in the association between caregiver-child interaction and neurodevelopmental scores

CpGs	Gene Symbol	Chr	Model 1		Model 2			Model 3			Model 4	
			β	*Nominal P value*	β	*Norminal P value*	*P*_FDR_	β	*Nominal P value*	*P*_FDR_	β	*Nominal P value*
cg06261610	DENND4B	1	0.526	0.010	-0.003	3.69E-04	0.899	0.875	8.50E-05	0.071	0.217	0.093
cg21055197	TMPRSS7	3	0.526	0.010	0.001	0.001	0.916	0.838	9.40E-05	0.071	0.061	0.058
cg07740897	CFAP45	1	0.526	0.010	-0.003	0.008	0.952	0.757	1.21E-04	0.071	0.101	0.057
cg07111566	FCGR3A	1	0.526	0.010	-0.001	0.001	0.899	0.856	1.49E-04	0.071	0.126	0.053
cg20666533	PCDH9	13	0.526	0.010	-0.002	0.005	0.951	0.794	1.81E-04	0.071	0.107	0.053
cg14447608	LAMC3	9	0.526	0.010	-0.002	0.004	0.945	0.803	2.17E-04	0.071	0.129	0.060
cg08665193	SMOC2	6	0.526	0.010	-0.001	0.013	0.952	0.755	2.21E-04	0.071	0.117	0.051
cg08482080	NANOS3	19	0.526	0.010	0.001	0.004	0.945	0.803	2.33E-04	0.071	0.192	0.052
cg13192640	FAM19A5	22	0.526	0.010	-0.002	0.006	0.952	0.791	2.58E-04	0.071	0.087	0.097
cg00608534	RAD51	15	0.526	0.010	0.0002	0.024	0.963	0.719	2.70E-04	0.071	0.125	0.051
cg05621113	PTPRN	2	0.526	0.010	-0.0002	0.009	0.952	0.776	3.03E-04	0.071	0.112	0.051
cg09071556	PRKG1	10	0.526	0.010	0.001	0.025	0.965	0.729	3.13E-04	0.071	0.103	0.062
cg07546710	SUN2	22	0.526	0.010	-0.0002	0.002	0.918	0.815	3.41E-04	0.071	0.056	0.051
cg27297326	C2	6	0.526	0.010	-0.001	0.008	0.952	0.779	3.64E-04	0.071	0.099	0.079
cg05151739	PLEKHG5	1	0.526	0.010	-0.0003	0.038	0.974	0.701	3.76E-04	0.071	0.133	0.093
cg08084502	CPEB1	15	0.526	0.010	0.002	0.008	0.952	0.781	3.77E-04	0.071	0.091	0.051
cg18086761	TDRKH	1	0.526	0.010	-0.001	0.027	0.968	0.736	3.93E-04	0.071	0.167	0.106
cg10142396	RUNX2	6	0.526	0.010	-0.001	0.031	0.968	0.728	4.00E-04	0.071	0.081	0.057
cg09189322	TCERG1	5	0.526	0.010	0.0002	0.035	0.971	0.722	4.14E-04	0.071	0.106	0.067
cg08075506	MTRR	5	0.526	0.010	0.001	0.021	0.960	0.751	4.46E-04	0.071	0.165	0.071

Chr: Chromosome. Model 1 assesses the association of caregiver-child interaction with children’s neurodevelopment; Model 2 examines the association of caregiver-child interaction with epigenome-wide DNA methylation level; Model 3 explores the association of epigenome-wide DNA methylation level with children’s neurodevelopment; Model 4 investigates the association between caregiver-child interaction and children’s neurodevelopment while adjusting the epigenome-wide DNA methylation level

## Discussion

In this epigenome-wide DNA methylation study utilizing an advanced 850k chip among vulnerable children from rural China, we identified a total of 844 CpG sites associated with ECD after FDR correction; interestingly, no CpG sites were found to be directly associated with caregiver-child interaction. Furthermore, our mediating analysis revealed that 395 CpG sites may mediate the association between caregiver-child interaction and children’s ECD before FDR correction. The findings from our study indicate that DNA methylation changes during early childhood may play a vital role in mediating the association of caregiver-child interaction and ECD, highlighting the potential significance of epigenetic mechanism as a target for early parenting intervention aimed at promoting the ECD of vulnerable children, particularly those experiencing poor caregiver-child interaction.

Consistent with findings from published studies, our research established a significant association between DNA methylation and ECD in vulnerable children from rural China. Among the top 20 genes annotated by significant CpG sites, several have been previously reported in the context of neuropsychological development and related disorders, including autism spectrum disorders, growth and development delays, and cognitive deficits. One noteworthy gene identified in our study is *CLN8*, which encodes a transmembrane endoplasmic reticulum (ER) protein that plays a crucial role in regulating the trafficking of catabolic enzymes from the ER to the Golgi, destined for the lysosome. CLN8 is significantly expressed in both developing and mature brains, and it is vital for the maturation, differentiation, and survival of various neuronal populations [36]. Furthermore, mutations in *CLN8* have been linked to neuronal ceroid lipofuscinoses (NCL), a prevalent neurodegenerative disease in childhood. NCL is characterized by symptoms such as myoclonus, tonic-clonic seizures, progressive declines in cognitive and motor function, and premature death [37]. In addition, *MCCC1* was also found to be associated with neurodevelopment. For example, a case-control study of 1428 Han Chinese reported that the allele of MCCC1/LAMP3 (rs11711441) polymorphism is linked to a lower risk of Parkinson’s disease [38]. However, previous studies involving the above-mentioned genes mainly focused on the association of gene mutations or polymorphisms with the nervous system and related diseases in clinical patients. Our study expanded this understanding by exploring the association between DNA methylation of these genes and neurodevelopment in the general population without clinical symptoms, suggesting that epigenetic changes may exert an important biological role in early neurodevelopment.

Several previous studies have reported the association between caregiver-child interaction and DNA methylation [16, 39]. However, our study found no CpG sites associated with caregiver-child interaction after FDR correction. This finding is consistent with a recently published epigenome-wide study conducted among 235 children from the Generation R Study. The absence of significant CpG site-level associations may suggest that such associations are subtle and challenging to detect, especially since our study focused on typical variation in caregiver-child interaction rather than extreme deviations (such as maltreatment) [40]. Besides, the sample size in our study may impede the detection of significant CpG sites after a stringent multiple-testing correction, emphasizing the potential benefits of exploring in larger samples. Despite the lack of significance after correction, we identified 33,565 caregiver-child interaction-associated CpG sites before FDR correction, providing insights into methylated signatures related to caregiver-child interaction. In addition, among the top 20 genes annotated by CpG sites associated with caregiver-child interaction, some genes, such as the *KCNG2* gene and *CACNA1C* gene, have been previously reported to be associated with ECD. Specifically, Haertle et al., found that some CpG sites in the *KCNG2* gene exhibited significant methylation differences between T21 cases and controls [41]. In addition, an epigenome-wide meta-analysis of four brain sample cohorts found that several methylated CpG sites in the *CACNA1C* gene were significantly associated with the Alzheimer’s disease (AD) Braak stage [42]. In this study, we also observed a relatively high inflation, as indicated by the inflation factor lambda. We hypothesize that unmeasured confounding exposures may have contributed to this issue. Future research, involving larger and more diverse cohorts with more comprehensive covariate adjustments, could mitigate inflation and enhance the robustness of the findings.

Early caregiving experience can profoundly affect offspring physiology, neural development, and behavior [43, 44]. Although the association of poor caregiver-child interaction with impaired ECD has been extensively explored, the underlying mechanism that drives this effect remains elusive. In our study focusing on preschool children in rural China, we sought to assess the potential mediating role of DNA methylation in the association between caregiver-child interaction and ECD. Utilizing CIT, our findings revealed that 395 CpG sites exhibited a significant mediating effect before FDR correction. Among the genes with the top 20 CpG sites, approximately half have been reported to be associated with neurodevelopment and related diseases. These genes include *CFAP45*,* PCDH9*,* LAMC3*,* FAM19A5*,* PRKG1*,* PLEKHG5*,* TCERG1*, and *MTRR.* For example, an epigenome-wide DNA methylation study in soldiers found an association between post-traumatic stress disorder and the DNA methylation of the *CFAP45* gene [45]. Moreover, a meta-analysis of genome-wide association studies highlighted the significant association of the single-nucleotide polymorphism (SNP) rs9540720 in the *PCDH9* gene with major depression disorder and cognitive function impairment [46]. In addition, the *LAMC3* gene mutation has been linked to severe bilateral smoothening and thickening of the lateral occipital cortex, impacting human brain structure and perceptual abilities [47]. FAM19A5, a secretory protein primarily expressed in the brain, is associated with neurological and psychiatric diseases. Mouse studies have revealed a role for *Fam19a5* in early nervous system development, with increased expression observed in response to pathological conditions in subsets of neurons and oligodendrocyte precursor cells of the adult traumatic brain [48]. A study with late-onset Alzheimer’s disease samples indicated a significant association of rs10824310 in the *PRKG1* gene [49]. *PLEKHG5* is a nuclear factor-κ-B-activator gene that predominantly expresses in the neurons and Schwann cells of the peripheral nervous system. A clinical case study of a young girl observed that the *PLEKHG5* mutations were related to lower motor neuron disease with dysmyelination in peripheral nerves [50]. To sum up, the genes annotated by some potential mediating CpG sites identified in our study have been previously reported to be involved in nervous system development. However, most earlier studies focused on gene polymorphism in animals and clinical adult patients. Our study, centered on vulnerable children aged 3–6 years, revealed a possible epigenetic mechanism linking poor caregiver-child interaction to ECD delay. This provides biological evidence supporting interventions for ECD in resource-constrained areas and populations. Our study highlighted the need for the pursuit of larger sample studies and the use of additional methods to further validate our results and provide a more comprehensive understanding of the role of DNA methylation in the associations between caregiver-interaction and ECD.

### Strengths and limitations

While the disproportionate burden of disease falls on marginalized populations, most studies are conducted in populations of European ancestry. Our study contributes to addressing the significant gap in research representation by focusing on preschool children from a low-resource rural area in China. Our investigation offers valuable and comprehensive insights into peripheral blood epigenome-wide DNA methylation changes associated with ECD using an advanced 850k chip, filling a crucial void in current epigenetic research that often relies on candidate gene methylation in the Western population. Moreover, we go beyond this by exploring the role of DNA methylation in the association between caregiver-child interaction and ECD. This expands the scope of current epigenetic studies and provides potential mechanisms linking caregiver-child interaction to ECD. Our research, conducted in a non-Western context, provides a theoretical basis for identifying new targets for early intervention. Several limitations should be mentioned in our study. First, the comparatively small sample size may limit the statistical power, potentially resulting in an underestimation of the methylated sites associated with ECD and caregiver-child interaction. Second, we acknowledge the inherent limitations of our case-control study design, particularly its inability to establish causal inferences. Future prospective cohort EWAS, especially those with larger sample sizes, have the potential to address these limitations. Third, we recognized the absence of the validation study in our research. While the genes with methylated sites identified in our research have been reported to be associated with neurological and/or psychiatric diseases, external validation is crucial to confirm the reliability of our results. Fourth, our study utilized CIT to infer a mediating role of DNA methylation in the relationship between caregiver-child interaction and ECD, which suggests but does not confirm a causal link. Consequently, our findings should be interpreted with caution. Future research may benefit from the integration of Mendelian Randomization approaches to strengthen the evidence for causality. Five, we were unable to further correlate the identified CpGs methylation and downstream expression in our study due to the lack of well-established database about methylation-gene expression for Chinese populations. Future research aimed at establishing these correlations will significantly enhance our understanding regarding the molecular mechanism underlying our observations.

## Conclusions

The early development of preschool children in Chinese low-resource areas is characterized by epigenome-wide DNA methylation changes. Importantly, these changes may play a mediation role in the association between poor caregiver-child interaction and delayed ECD. Our findings contribute evidence of epigenetic associations in the context of early development and underscore the potential role of parenting interventions in supporting disadvantaged children. However, the results warrant further validation across diverse populations.

## Electronic supplementary material

Below is the link to the electronic supplementary material.

Supplementary Material 1

## Data Availability

Data is available from the corresponding author through reasonable request.

## Abbreviations

ECD: Early childhood development
EWAS: Epigenome-wide association study
DQ: Developmental quotient
CIT: Causal inference test
NDS: Neurodevelopmental score
GDDS: The Gesell Developmental Diagnostic Scale
BPCIS: The Chinese version of Brigance Parent-Child Interactions Scale
FDR: False-Discovery-Rate
AD: Alzheimer’s disease
