# Supplementary material for: Caregiver-child interaction and early childhood development among preschool children in rural China: the possible role of blood epigenome-wide DNA methylation
Source: BMC Genomics. 2025 Apr 1;26:329. doi: 10.1186/s12864-025-11406-2 (PMC11963332; doi:10.1186/s12864-025-11406-2)
Supplement: Supplementary file 1 — Supplementary Material 1 [file 12864_2025_11406_MOESM1_ESM.docx]

**Caregiver-child interaction and** **early childhood development among** **preschool children in rural China:** **the possible role of blood epigenome-wide DNA methylation**

Mengna Wei ^a*^, Rui Chang ^a,b*^, Chunan Li ^a^, Yanfen Jiang ^a^, Jianduan Zhang ^a,c^

Affiliation: ^a^ Department of Maternal and Child Health, School of Public Health, Tongji Medical College, Huazhong University of Science and Technology, Wuhan, China

^b^ Division of Child Healthcare, Department of Pediatrics, Tongji Hospital, Tongji Medical College, Huazhong University of Science and Technology, Wuhan, China

^c^ Key Laboratory of Environment and Health, Ministry of Education and Ministry of Environmental Protection, State Key Laboratory of Environmental Health (Incubating), School of Public Health, Tongji Medical College, Huazhong University of Science and Technology, 13 Hangkong Road, Wuhan, Hubei, 430030, China

*Mengna Wei and Rui Chang contributed equally to this work.

Address correspondence to: Jianduan Zhang, School of Public Health, Tongji Medical College, Huazhong University of Science and Technology, Wuhan, China, [jd_zh@hust.edu.cn], 027-83692755

Table S1 Characteristics of the included and non-included participants

| Variables | Characteristics | Included  (n=64),  n %(n) | Not included  (n=107),  n %(n) | *χ^2^/t* | *P* |
| --- | --- | --- | --- | --- | --- |
| Sex of child | Boy | 56.2 (36) | 50.5 (54) | 0.537 | 0.464 |
| Mode of Delivery | Vaginal delivery | 34.4 (22) | 30.8 (33) | 0.221 | 0.630 |
|  | Cesarean section | 65.6 (42) | 69.2(74) |  |  |
| Preterm | Yes | 12.5(8) | 10.2(11) | 0.205 | 0.652 |
| Non-only child | Yes | 75.0 (48) | 71.9(77) | 0.181 | 0.676 |
| Feeding mode in the first six months | Basic exclusive breastfeeding | 50.0(32) | 53.3(57) | 0.218 | 0.901 |
|  | Mixed feeding | 35.9(23) | 32.7(35) |  |  |
|  | Formula feeding | 14.1(9) | 14.0(15) |  |  |
| Paternal education level | Primary school or less | 6.3(4) | 9.3(10) | 0.525 | 0.770 |
|  | Junior/senior/vocational secondary school | 87.5(56) | 85.0(91) |  |  |
|  | College or more | 6.3(4) | 5.6(6) |  |  |
| Maternal education | Primary school or less | 12.5(8) | 16.8(18) | 3.043 | 0.211 |
|  | Junior/senior/vocational secondary school | 85.9(55) | 76.6(82) |  |  |
|  | College or more | 1.6(1) | 6.5(7) |  |  |
| Household monthly income (Yuan) | ＜2000 | 20.3 (13) | 23.3(25) | 0.656 | 0.883 |
|  | 2000~3999 | 37.5(24) | 31.7(34) |  |  |
|  | 4000-5999 | 21.9(14) | 24.3(26) |  |  |
|  | ≥6000 | 20.3(13) | 20.7 (22) |  |  |
| Global NDS | - | 86.7 ± 17.0 | 89.9 ± 7.5 | -0.454 | 0.638 |

Notes: Global NDS: global neurodevelopmental score.

| Table S2 The top 10 KEGG pathways for gene annotated by CpGs associated with global NDS | | | | |
| --- | --- | --- | --- | --- |
| ID | Pathways | No. genes | Nominal p value | *P*_FDR_-value |
| hsa04921 | Oxytocin signaling pathway | 147 | 9.40E-09 | 7.85E-07 |
| hsa04150 | mTOR signaling pathway | 146 | 5.93E-07 | 1.26E-05 |
| hsa04919 | Thyroid hormone signaling pathway | 115 | 9.05E-07 | 1.61E-05 |
| hsa05022 | Pathways of neurodegeneration | 417 | 9.15E-07 | 1.61E-05 |
| hsa04912 | GnRH signaling pathway | 90 | 1.08E-06 | 1.71E-05 |
| hsa04024 | cAMP signaling pathway | 201 | 2.07E-06 | 3.01E-05 |
| hsa04728 | Dopaminergic synapse | 124 | 2.43E-06 | 3.25E-05 |
| hsa04722 | Neurotrophin signaling pathway | 112 | 5.82E-06 | 6.71E-05 |
| hsa04390 | Hippo signaling pathway | 145 | 6.12E-06 | 6.81E-05 |
| hsa04014 | Ras signaling pathway | 209 | 7.54E-06 | 7.94E-05 |

Note: No. genes mean the number of genes. Global NDS: global neurodevelopmental score

| Table S3 The top 10 GO pathways for gene annotated by CpGs associated with global NDS | | | | | |
| --- | --- | --- | --- | --- | --- |
| Ontology | ID | Function | No. genes | Nominal p value | *P*_FDR_-value |
| Biological processes (BP) | GO:0060284 | Regulation of cell development | 438 | 3.38E-17 | 6.81E-14 |
|  | GO:0010975 | Regulation of neuron projection development | 390 | 4.29E-17 | 6.81E-14 |
|  | GO:0001558 | Regulation of cell growth | 368 | 2.87E-15 | 2.60E-12 |
|  | GO:0051960 | Regulation of nervous system development | 377 | 3.25E-13 | 1.63E-10 |
|  | GO:0016311 | Dephosphorylation | 434 | 3.34E-13 | 1.63E-10 |
|  | GO:0097485 | Neuron projection guidance | 256 | 4.83E-13 | 2.05E-10 |
|  | GO:0048167 | Regulation of synaptic plasticity | 167 | 5.02E-12 | 1.33E-09 |
|  | GO:0050767 | Regulation of neurogenesis | 311 | 3.54E-11 | 6.62E-09 |
|  | GO:0048880 | Sensory system development | 327 | 1.37E-10 | 2.07E-08 |
|  | GO:0009896 | Positive regulation of catabolic process | 393 | 2.14E-10 | 3.15E-08 |
| Cellular  Component (CC) | GO:0005911 | Cell-cell junction | 439 | 1.09E-17 | 8.31E-15 |
|  | GO:0098793 | Presynapse | 436 | 1.81E-15 | 4.61E-13 |
|  | GO:0043025 | Neuronal cell body | 423 | 1.76E-14 | 2.68E-12 |
|  | GO:0030055 | Cell-substrate junction | 380 | 3.37E-14 | 4.28E-12 |
|  | GO:0098984 | Neuron to neuron synapse | 306 | 3.61E-11 | 2.75E-09 |
|  | GO:0098687 | Chromosomal region | 308 | 5.95E-11 | 4.13E-09 |
|  | GO:0099568 | Cytoplasmic region | 234 | 2.94E-10 | 1.44E-08 |
|  | GO:0031253 | Cell projection membrane | 299 | 6.18E-10 | 2.48E-08 |
|  | GO:0000775 | chromosome, centromeric region | 181 | 7.01E-10 | 2.68E-08 |
|  | GO:0044309 | Neuron spine | 164 | 7.18E-09 | 1.89E-07 |
| Molecular function (MF) | GO:0045296 | Cadherin binding | 310 | 7.17E-14 | 8.63E-11 |
|  | GO:0004674 | Protein serine/threonine kinase activity | 394 | 1.43E-13 | 8.63E-11 |
|  | GO:0016887 | ATPase activity | 434 | 4.17E-13 | 1.68E-10 |
|  | GO:0005543 | Phospholipid binding | 403 | 8.16E-10 | 2.47E-07 |
|  | GO:0046873 | Metal ion transmembrane transporter activity | 379 | 4.81E-09 | 8.31E-07 |
|  | GO:0030674 | Protein-macromolecule adaptor activity | 241 | 8.13E-09 | 1.23E-06 |
|  | GO:0042578 | Phosphoric ester hydrolase activity | 329 | 1.24E-08 | 1.67E-06 |
|  | GO:0060090 | Molecular adaptor activity | 306 | 6.52E-08 | 7.88E-06 |
|  | GO:0003712 | Transcription coregulator activity | 421 | 1.04E-07 | 1.09E-05 |
|  | GO:0106310 | Protein serine kinase activity | 226 | 2.02E-07 | 1.63E-05 |

Note: No. genes mean the number of genes. Global NDS: global neurodevelopmental score.

| Table S4 The top 10 KEGG pathways for gene annotated by CpGs associated with caregiver-child interaction | | | | | |  |
| --- | --- | --- | --- | --- | --- | --- |
| ID | Pathways | No. genes | Nominal p value | *P*_FDR_-value |  |  |
| hsa04360 | Axon guidance | 137 | 1.95E-10 | 6.52E-08 |  |  |
| hsa04510 | Focal adhesion | 144 | 2.11E-08 | 3.53E-06 |  |  |
| hsa04144 | Endocytosis | 172 | 1.59E-07 | 1.77E-05 |  |  |
| hsa04310 | Wnt signaling pathway | 119 | 5.70E-07 | 4.76E-05 |  |  |
| hsa05205 | Proteoglycans in cancer | 142 | 7.29E-07 | 4.87E-05 |  |  |
| hsa05165 | Human papillomavirus infection | 216 | 1.54E-06 | 8.56E-05 |  |  |
| hsa04928 | Parathyroid hormone synthesis, secretion and action | 79 | 2.79E-06 | 0.00013 |  |  |
| hsa04010 | MAPK signaling pathway | 192 | 5.47E-06 | 0.00023 |  |  |
| hsa04015 | Rap1 signaling pathway | 141 | 1.16E-05 | 0.00036 |  |  |
| hsa05100 | Bacterial invasion of epithelial cells | 59 | 1.18E-05 | 0.00036 |  |  |

Note: No. genes mean the number of genes

| Table S5 The top 10 GO pathways for gene annotated by CpGs associated with caregiver-child interaction | | | | | |
| --- | --- | --- | --- | --- | --- |
| Ontology | ID | Function | No. genes | Nominal *p* value | *P*_FDR_-value |
| Biological processes (BP) | GO:0007409 | Axonogenesis | 316 | 3.04E-18 | 1.93E-14 |
|  | GO:0010975 | Regulation of neuron projection development | 295 | 1.98E-17 | 6.28E-14 |
|  | GO:0031589 | Cell-substrate adhesion | 252 | 1.33E-16 | 2.82E-13 |
|  | GO:0099177 | Regulation of trans-synaptic signaling | 279 | 2.74E-16 | 4.34E-13 |
|  | GO:0050804 | Modulation of chemical synaptic transmission | 278 | 3.89E-16 | 4.93E-13 |
|  | GO:0090066 | Regulation of anatomical structure size | 328 | 9.90E-16 | 1.05E-12 |
|  | GO:0045785 | Positive regulation of cell adhesion | 288 | 1.60E-15 | 1.45E-12 |
|  | GO:0007015 | Actin filament organization | 292 | 6.74E-15 | 5.34E-12 |
|  | GO:0032535 | Regulation of cellular component size | 253 | 1.68E-14 | 1.18E-11 |
|  | GO:0097485 | Neuron projection guidance | 198 | 2.43E-14 | 1.54E-11 |
| Cellular Component (CC) | GO:0031252 | Cell leading edge | 291 | 8.06E-20 | 6.12E-17 |
|  | GO:0030055 | Cell-substrate junction | 288 | 6.31E-16 | 2.40E-13 |
|  | GO:0005925 | Focal adhesion | 283 | 1.33E-15 | 3.38E-13 |
|  | GO:0098793 | Presynapse | 318 | 1.21E-13 | 2.30E-11 |
|  | GO:0005911 | Cell-cell junction | 316 | 2.20E-13 | 3.35E-11 |
|  | GO:0005938 | Cell cortex | 207 | 1.75E-12 | 2.21E-10 |
|  | GO:0001726 | Ruffle | 127 | 1.42E-10 | 1.54E-08 |
|  | GO:0005874 | Microtubule | 271 | 1.64E-10 | 1.56E-08 |
|  | GO:0014069 | Postsynaptic density | 208 | 2.11E-10 | 1.78E-08 |
|  | GO:0030027 | Lamellipodium | 139 | 2.82E-10 | 1.96E-08 |
| Molecular function (MF) | GO:0003779 | Actin binding | 298 | 1.81E-13 | 2.19E-10 |
|  | GO:0004674 | Protein serine/threonine kinase activity | 289 | 2.60E-12 | 1.57E-09 |
|  | GO:0045296 | Cadherin binding | 226 | 1.00E-10 | 4.03E-08 |
|  | GO:0030695 | GTPase regulator activity | 297 | 1.19E-08 | 3.60E-06 |
|  | GO:0030674 | Protein-macromolecule adaptor activity | 178 | 1.84E-08 | 3.89E-06 |
|  | GO:0051015 | Actin filament binding | 145 | 2.08E-08 | 3.89E-06 |
|  | GO:0060090 | Molecular adaptor activity | 224 | 2.25E-08 | 3.89E-06 |
|  | GO:0035091 | Phosphatidylinositol binding | 171 | 4.04E-08 | 6.11E-06 |
|  | GO:0004713 | Protein tyrosine kinase activity | 99 | 7.03E-08 | 8.70E-06 |
|  | GO:0003712 | Transcription coregulator activity | 301 | 7.19E-08 | 8.70E-06 |

Note: No. genes mean the number of genes
